# 

**Published:** 1876-07

**Authors:** 


### CONTENTS.

PAGE.

**CONTRIBUTIONS.**

Compromises with Nature. BEN. H. PRATT............141
Paralysis of the Stomach. P. H. HAYES, M. D........143
Doctors. DR. NED............144
The Fly. H............146

**CLIPPINGS.**

The Danger of Flowering and Fruit-bearing Plants in Sleeping Apartments—To Extirpate House Insects—The Making of Doctors—Soft Hands............147

**MEDICAL ITEMS AND NEWS.**

Hiccough—Herpes Zoster—Goddard's Cosmetic Lotion—Grapes in Fever—A Hard Case—Anti-Rheumatic Mixture—Offensiveness of Breath and Expectoration—Delirium Tremens—Cold in the Head—Enemata of Chloral—Hydrophobia—Pityriasis—The Eucalyptus—Ague—Neuralgia—Detecting Sugar in Urine—Skin Diseases—Rheumatism—Mead—Tea Poultice—Specific against Hydrophobia—Typhoid Fever—Diabetes Millitus—Medicated Ice—Bowel Affections—Otorrhœa—Fancy Soda Water Syrups—Diabetes Treated by Skimmed Milk—Rapid Cure of Acute Rheumatism with Salicylic Acid............148 to 154

**OUR CORRESPONDENCE.**

Florida Letter. S. O. GLEASON, M. D............155
"The Great English Physician" again............156
Four Letters with Replies............156 to 159

PAGE.

**HOUSEHOLD HINTS AND RECIPES.**

Paste for Tin—Diamond Cement—To Remove Wax Spots—Cement—Ivory, to Render Ductile—Jelly Water—A Hint for the Laundry—Lotions for Freckles—How to Remove Stains from Steel Knives—Condensed Eggs—For Frost-Bite—Mucilage for Mineralogical Specimens—To Remove Grease Spots—To polish Tins—Cement for Marble and Alabaster—Gas from Petroleum—Water Proof Paint for Canvas—Whitewash—Coating for Black Boards—Gouland's Lotion—Silvering Glass—Curry Powder—Cure for Love of Liquor—Painting Old Buildings—Water-Proof Glue—Fruit Eating—Copying Ink Requiring no Press—Gilding—To Furbish Oil Cloths—To Remove Grease and Other Spots—Scouring Liquid—Feeding Babies with Starch............160 to 163

**EDITORIAL.**

Spectacles............164
Anchylosis............164

**EDITORIAL CHIT-CHAT.**

Florida Letter—The Floridians—Five-Year Olds—H. at Last—Trichina—Photographing Extraordinary—Mr. Towner's Poem—Left Over—Camping Out—Compromises with Nature—Our Driving Park—Flowers—The Centennial—Thrush—Faithful Officials—American Girl Statue—Valuable Cross—Plumbers' Help—Builders' Estimates—It makes a Difference............166 to 168

